# Populations of *Helicoverpa zea* (Boddie) in the Southeastern United States are Commonly Resistant to Cry1Ab, but Still Susceptible to Vip3Aa20 Expressed in MIR 162 Corn

**DOI:** 10.3390/toxins13010063

**Published:** 2021-01-15

**Authors:** Ying Niu, Isaac Oyediran, Wenbo Yu, Shucong Lin, Marcelo Dimase, Sebe Brown, Francis P. F. Reay-Jones, Don Cook, Dominic Reisig, Ben Thrash, Xinzhi Ni, Silvana V. Paula-Moraes, Yan Zhang, Jeng Shong Chen, Zhimou Wen, Fangneng Huang

**Affiliations:** 1 Department of Entomology, Louisiana State University Agricultural Center, Baton Rouge, LA 70803, USA; YNiu@agcenter.lsu.edu (Y.N.); wyu14@lsu.edu (W.Y.); slin9@lsu.edu (S.L.); msdimase@agcenter.lsu.edu (M.D.); 2 Syngenta Crop Protection LLC, Research Triangle Park, NC 27709, USA; isaac.oyediran@syngenta.com (I.O.); yan.zhang@syngenta.com (Y.Z.); eric.chen@syngenta.com (J.S.C.); zhimou.wen@syngenta.com (Z.W.); 3 Dean Lee Research Station, Louisiana State University Agricultural Center, Alexandria, LA 71302, USA; SBrown@AgCenter.lsu.edu; 4 Department of Plant and Environmental Sciences, Clemson University, Florence, SC 29506, USA; freayjo@clemson.edu; 5 Delta Research and Extension Center, Mississippi State University, Stoneville, MS 38776, USA; DCook@drec.msstate.edu; 6 Vernon G. James Research and Extension Center, North Carolina State University, Plymouth, NC 27962, USA; ddreisig@ncsu.edu; 7 Lonoke Extension Center, University of Arkansas, Lonoke, AR 72086, USA; bthrash@uaex.edu; 8 Crop Genetics and Breeding Research, USDA-ARS, Tifton, GA 3173, USA; xinzhi.ni@usda.gov; 9 Entomology & Nematology Department, West Florida Research and Education Center, University of Florida, Jay, FL 32565, USA; paula.moraes@ufl.edu

**Keywords:** corn earworm, Bt susceptibility, Cry1Ab, Vip3A, resistance monitoring

## Abstract

The corn earworm, *Helicoverpa zea* (Boddie), is a major pest targeted by pyramided *Bacillus thuringiensis* (Bt) corn and cotton in the U.S. Cry1Ab is one of the first insecticidal toxins used in Bt crops, while Vip3A is a relatively new toxin that has recently been incorporated into Cry corn with event MIR 162 and Cry cotton varieties to generate pyramided Bt traits targeting lepidopteran pests including *H. zea*. The objectives of this study were to determine the current status and distribution of the Cry1Ab resistance, and evaluate the susceptibility to Vip3Aa20 expressed in MIR 162 corn in *H. zea* in the southeastern U.S. During 2018 and 2019, 32 *H. zea* populations were collected from non-Bt corn (19 populations), Cry corn (12), and Cry/Vip3A cotton (1) across major corn areas in seven southeastern states of the U.S. Susceptibility of these populations to Cry1Ab and Vip3Aa20 was determined using diet-overlay bioassays. Compared to a known susceptible insect strain, 80% of the field populations were 13- to >150-fold resistant to Cry1Ab, while their response to Vip3Aa20 ranged from >11-fold more susceptible to 9-fold more tolerant. Mean susceptibility to each Bt toxin was not significantly different between the two groups of the populations collected from non-Bt and Bt crops, as well as between the two groups of the populations collected during 2018 and 2019. The results show that resistance to Cry1Ab in *H. zea* is widely distributed across the region. However, the Cry1Ab-resistant populations are not cross-resistant to Vip3Aa20, and *H. zea* in the region is still susceptible to the Vip3Aa20 toxin. Vip3Aa20 concentrations between 5 and 10 µg/cm^2^ may be used as diagnostic concentrations for susceptibility monitoring in future. Additional studies are necessary to elucidate the impact of the selection with Bt corn on resistance evolution in *H. zea* to Vip3A cotton in the U.S.

## 1. Introduction

The corn earworm, *Helicoverpa zea* (Boddie), is a cross-crop target pest of both *Bacillus thuringiensis* (Bt) corn and cotton in the U.S. [1,2]. Corn and cotton are usually planted in close proximity in the southern U.S. (often called the U.S. Cotton Belt) where predominant areas of the two crops are planted with varieties containing Bt transgenes [3]. The typical host progression of *H. zea* in the southern U.S. is that the first generation develops on wild hosts, the second generation primarily feeds on corn, and the subsequent 2 to 3 generations switch to cotton and other hosts before overwintering [2,4]. In addition, some of the populations originating from the Cotton Belt migrate into the northern regions where they can cause damage to many crops and vegetables [5]. Because Bt toxins produced in transgenic corn and cotton are similar, *H. zea* in the southern U.S. is subjected to resistance selection for multiple generations each year. The cropping system plus the similarity of Bt toxins expressed in corn and cotton creates an ideal environment for resistance development in *H. zea* [1,4]. 

During recent years, widespread field resistance to Cry1A.105/Cry2Ab corn and Cry1Ac/Cry2Ab cotton in *H. zea* has been documented in the U.S. [1,6,7,8,9]. In its early commercial use, the single-gene Cry1Ab corn (e.g., YieldGard^®^ Corn Borer) was marginally effective against *H. zea* [10,11]. By 2012–2013, field trials in North and South Carolina showed that Cry1Ab corn was no longer efficacious against *H. zea* and the control failures were believed to be most likely due to resistance development [12]. In Louisiana, Cry1Ab corn also failed to control *H. zea* during 2017 (FH, unpublished data, personal communication). Unfortunately, there were no follow-up studies to further document the Cry1Ab resistance. 

Vip3A is an insecticidal toxin produced during the vegetative growth stages of Bt bacteria [13]. Compared to Cry toxins, Vip3A has a distinct mode of action and thus cross-resistance between Vip3A and Cry toxins is unlikely [13,14,15,16,17,18]. Vip3A, as two variants of a native Vip3Aa1, was first registered for commercial use in Bt cotton (e.g., VipCot™ cotton) in 2008 and Bt corn (e.g., MIR 162) in 2011. The Vip3A gene used in cotton is Vip3Aa19, while it is Vip3Aa20 in corn [19,20]. Vip3Aa19 and Vip3Aa20 are very similar in gene structure, both sharing >99.7% amino acid sequence identity with the native toxin and having the same mode of action [19]. Corn and cotton varieties expressing Vip3A toxin are highly effective in *H. zea* control [21,22,23,24,25]. However, the areas planted to Vip3A-expressing crops had been limited until recently [26]. The widespread occurrence of Cry1/Cry2 resistance in *H. zea* greatly encourages the use of Vip3A crops, especially in cotton in the U.S. Additionally, field resistance of the fall armyworm, *Spodoptera frugiperda* (J.E. Smith), to Cry1F corn has become widespread in the Americas [26,27,28,29,30]. Vip3A corn has also become an important tool for managing the Cry1F-resistant *S. frugiperda*, especially in South America [31]. Therefore, preservation of the susceptibility to Vip3A in these target pests is crucial for the continued success of transgenic Bt plant technology.

Resistance monitoring of field insect populations is a fundamental component of effective resistance management programs [4,32]. Data on geographical susceptibility of pest populations are essential in resistance monitoring. Unfortunately, information on geographical susceptibilities of *H. zea* populations to Cry1Ab and Vip3A is limited, with only two peer-reviewed studies related to Cry1Ab susceptibility established before and during the early commercial use of Cry1A crops [33,34]. The only peer-reviewed study related to *H. zea* susceptibility to Vip3Aa20 is from Brazil [31]. Another early study in the U. S. evaluated the susceptibility of *H. zea* to Vip3Aa19 expressed in Bt cotton [20]. In the current study, 32 *H. zea* populations were collected from non-Bt corn (19 populations), Cry corn (12), and Cry/Vip3A cotton (1) fields across major corn production areas in seven states of southeastern U.S. Susceptibility of these populations to Cry1Ab and Vip3Aa20 was determined using diet-overlay bioassays [8]. The objectives of the current study were to determine the current status and geographic distribution of Cry1Ab resistance, and monitor Vip3Aa20 susceptibility in *H. zea* in the region. Information generated from this study will be useful in developing appropriate measures for mitigating the Cry resistance and improving current resistance management programs to sustain *H. zea* susceptibility to Vip3A.

## 2. Results

### 2.1. Resistance to Cry1Ab in Helicoverpa zea is Common Across the Southeastern United States 

**Figure 1:**
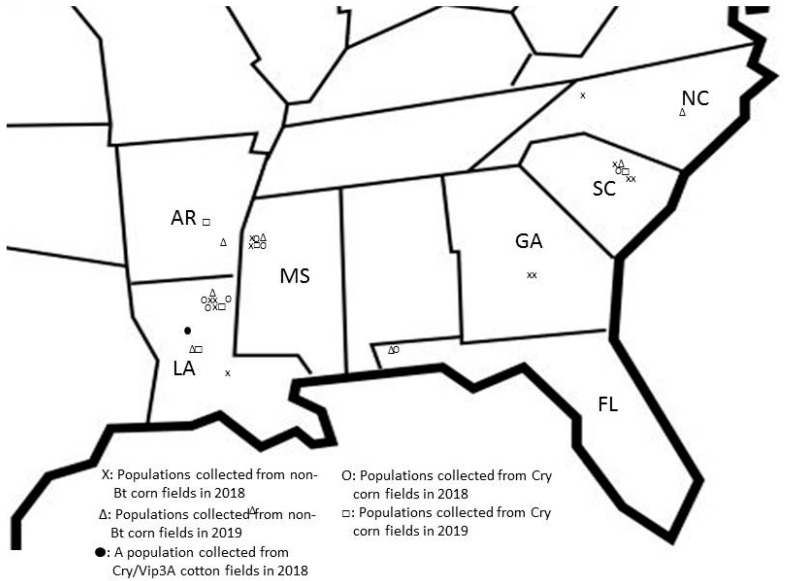
Collection sites of *Helicoverpa* *zea* populations in seven states of the southeastern U. S. during 2018 and 2019.

Cry1Ab susceptibility of F_1_ or F_2_ neonates of 30 field populations collected during 2018 (20 populations) and 2019 (10) was compared with that of a known Bt-susceptible laboratory strain (BZ) using diet-overlay bioassays. These field insect populations were collected from non-Bt corn (18 populations), Cry corn (11), and Cry/Vip3A cotton (1) fields (Figure 1; Table 1). The bioassays confirmed that BZ was susceptible to Cry1Ab with a median lethal concentration (LC_50_) of 0.21µg/cm^2^ and a 95% confidence interval (CI) of 0.15 to 0.29 (Table 1).

LC_50_ values of the 20 populations collected during 2018, which consisted of 12 populations from non-Bt and 8 from Bt crop fields, ranged from 1.84 to >31.6 µg/cm^2^ (Table 1). Compared to the LC_50_ value of the reference strain BZ, even the two populations (MS-ST-NBt-1 and FL-SR-VT2P) that showed the lowest LC_50_ values (1.84 µg/cm^2^) had a resistance ratio of 9-fold to Cry1Ab, and the 9-fold difference was significant based on the non-overlapped 95% CIs with that of BZ. Five populations had resistance ratios of 13- to 37-fold; the remaining 13 populations had ≥43-fold resistance (Table 1). At 10 µg/cm^2^, 10 of the 20 populations collected in 2018 exhibited >50% larval survival rates (Table 1). The average larval survivorship at 10 µg/cm^2^ was not significantly different between the group of the 12 populations collected from non-Bt crops (49.74% ± 6.4, mean ± SEM) and the group of the 7 populations from Bt crops (55.4 ± 3.3%) (Table 2) (Note, the survival data at 10 µg/cm^2^ for the population FL-SR-VT2P was not available).

Cry1Ab LC_50_ values for the 10 populations collected during 2019, which included 6 populations from non-Bt and 4 populations from Bt corn fields, ranged from 0.40 to >10 µg/cm^2^ (Table 1). Compared to the susceptibility of BZ, the LC_50_ values of the 10 populations equated to resistance ratios of 2- to >48-fold. The LC_50_ values of the two populations (AR-TR-NBt and FL-SR-NBt) that showed the lowest resistant ratio (2-fold) were not significantly different from the LC_50_ of BZ based on the overlapped 95% CIs with that of BZ. Two populations (LA-AX-VT2P and MS-ST-NBt-2) also showed a low level (5-fold) of resistance to Cry1Ab, but the 5-fold difference was significant based on the non-overlapped 95% CIs with that of BZ. Another two populations (MS-ST-VT2P-2 and SC-DL-VT2P-2) had resistance ratios of 11- and 29-fold, respectively. Resistance ratios for the remaining four populations were ≥40-fold. At 10 µg/cm^2^, the group of the six populations from non-Bt corn had a mean survival rate of 37.9 ± 13.3%, which was not significantly different compared to the mean (45.1 ± 12.1%) of the group of the four populations from Bt corn fields (Table 2).

Student’s t-tests also showed that the mean survivorship (40.8 ± 8.9%) of the group of the 10 populations collected during 2019 was not significantly different compared to that (51.8 ± 4.2%) of the group of the 19 populations collected during 2018 (Table 2). In addition, mean survivorship (45.8 ± 6.1%) at 10 µg/cm^2^ for the group of the 18 populations collected from non-Bt crops during the two years was also not significantly different from that (51.7 ± 4.7%) of the group of the 11 populations from Bt crop (Table 2).

### 2.2. Field Populations of Helicoverpa zea in the Southeastern United States Are Still Susceptible to Vip3Aa20

Vip3Aa20 susceptibility of F_1_ or F_2_ neonates of 29 field populations of *H. zea* collected during 2018 (18 populations) and 2019 (11), along with BZ, was assayed using the diet-overlay bioassays (Figure 1; Table 3). These field populations were collected from non-Bt corn (18 populations), Cry corn (10), and Cry/Vip3A cotton (1) fields. Twenty-seven of the 29 populations were also assayed against Cry1Ab (mentioned above). Probit analysis estimated the LC_50_ of BZ to be 0.40 µg Vip3Aa20/cm^2^ with a 95% CI of 0.30 to 0.51 (Table 3).

Compared to BZ, among the 18 field populations collected during 2018, which consisted of 12 populations from non-Bt and 6 from Bt corn fields, 10 populations had similar LC_50_ values as BZ based on their overlapping 95% CIs with that of BZ; six had significantly lower LC_50_ values, while two had significantly greater LC_50_ values (Table 3). Among the 18 populations, the most susceptible population (GA-TF-NBt-2) to Vip3Aa20 was >11.5-fold more sensitive than BZ. Susceptibility ratios relative to BZ of all other field populations, ranged from 3.1-fold more sensitive to 2.4-fold more tolerant (Table 3). Relative to BZ, the only population (LA-GT-WS3) collected from cotton plants containing Vip3Aa19 gene showed a 2.3-fold more tolerant to the Vip3Aa20 protein. The 2.3-fold difference was significant based on the non-overlapped 95% CIs of the LC_50_s with that of BZ, but the susceptibility of LA-GT-WS3 was well within the range of other field populations (Table 3). Probit analysis on the pooled bioassay data of the 12 populations collected from non-Bt corn showed an LC_99_ of 7.0 µg/cm^2^ with a 95% CI of 5.0 to 10.7. Student’s *t*-tests showed that the mean LC_50_ value (0.37 ± 0.08 µg/cm^2^) of the group of the 12 populations from non-Bt plants was similar to that (0.45 ± 0.10 µg/cm^2^) of the group of the 6 populations from Bt plants (Table 4).

Among the 11 populations collected during 2019, which included 6 from non-Bt and 5 from Bt corn fields, three had similar LC_50_ values as BZ; five had significantly lower LC_50_ values; and three had significantly greater LC_50_ values than BZ (Table 3). The susceptibility ratios of these 11 populations, relative to BZ, ranged from 10.0-fold more sensitive to 9.0-fold more tolerant (Table 3). Student’s *t*-tests showed that the mean LC_50_ value (0.53 ± 0.26 µg/cm^2^) of the group of the six populations from non-Bt plants was not significantly different to that (0.97 ± 0.67 µg/cm^2^) of the group of the five populations from Bt plants (Table 4).

In addition, t-tests also showed that the mean LC_50_ value (0.73 ± 0.32 µg/cm^2^) of the group of the 11 populations collected during 2019 was not significantly different from that (0.40 ± 0.06 µg/cm^2^) of the group of the 18 populations collected during 2018 (Table 4). Furthermore, the mean LC_50_ value (0.42 ± 0.10 µg/cm^2^) of the group of the 18 populations collected from non-Bt crops during the two years was also not significantly different from that (0.69 ± 0.30 µg/cm^2^) of the group of the 11 populations from Bt crop fields (Table 4).

### 2.3. There Are No Significant Relationships Between the Susceptibilities of Helicoverpa Zea Populations to Cry1Ab and Vip3Aa20 Proteins

Seventeen populations collected during 2018 and nine populations sampled during 2019 were assayed against both Cry1Ab and Vip3Aa20 in the study. Linear regression analysis showed that the larval survivorships (X) at 10 µg Cry1Ab/cm^2^ were not significantly correlated to the LC_50_ values (Y) of Vip3Aa20 for the 17 populations collected in 2018 (Y = 0.296 + 0.002 X; *R*^2^ = 0.024; *p* = 0.5524) and for the 9 populations collected in 2019 (Y = 0.045 + 0.008 X; *R*^2^ = 0.218; *p* = 0.2051), as well as for the full set of the 26 populations collected across both years (Y = 0.156 + 0.005X; *R*^2^ = 0.1086; *p* = 0.1003).

## 3. Discussion

Transgenic corn and cotton containing single Cry1 genes (e.g., Cry1Ac or Cry1Ab) were first commercialized in the U.S. in 1995/1996 [4]. The single-gene Cry1Ab corn hybrids did not provide a high dose against *H. zea*, but they were originally able to suppress the pest population and reduce ear damage [10,11]. Two studies reported that field populations of *H. zea* were relatively susceptible to Cry1Ab in the U.S. during the early commercial use of Cry1A crops [33,34]. The current study showed that, although considerable variations still existed among the populations evaluated, the majority of *H. zea* populations exhibited significant levels of resistance to Cry1Ab, and the overall resistance levels were consistent between the two groups of populations collected from non-Bt and Bt plants, as well as between the two groups of populations collected during 2018 and 2019. In particular, for the seven populations collected from North and South Carolina, the three least resistant populations exhibited 17- to 40-fold resistance to Cry1Ab, and the other four had resistance ratios of >48-fold. The results of the current study validated that the previous observations in the field showing a lack of significant impact of Cry1Ab corn on kernel injury and *H. zea* occurrence was due to resistance development in the insect [12]. The results of the current study further demonstrate that the resistance to Cry1Ab corn in *H. zea* has become widely distributed across the southeastern U.S. 

Ali and Luttrell [20] evaluated Vip3Aa19 susceptibility of 31 *H. zea* populations collected during 2004 to 2007 from the southern U.S.; susceptibility ratios relative to a laboratory strain ranged from 27-fold more susceptible to 2.2-fold more tolerant. The current study showed that the overall Vip3Aa20 susceptibility of *H. zea* populations collected during 2018 and 2019 was similar as that reported in the reference [20]. In addition, the regression analysis exhibited that there were no significant relationships between the Cry1Ab resistance levels and Vip3Aa20 susceptibilities for the populations collected in the current study. The results show that the Cry1Ab-resistant *H. zea* populations do not present cross-resistance to Vip3Aa20; and the populations in the region, regardless of their host plant sources (non-Bt and Bt crops), collection years (2018 and 2019), or their resistance levels to Cry1Ab, are still susceptible to the Vip3Aa20 toxin. An early study also showed that a Cry1Ac-selected *H. zea* population was not cross-resistant to Vip3A toxin [35], while another study reported that a Cry1Ac-selected *H. zea* strain was 1.6-fold less susceptible to Vip3A than the un-selected counterpart [36]. A recent review based on bioassays with several insect species targeted by Bt corps showed a weak cross-resistance pattern between Cry1 and Vip3 [37].

Based on the 95% CI of LC_99_ estimated from the pooled bioassay data with the 12 populations collected from non-Bt corn during 2018, we believe that Vip3Aa20 concentrations between 5 to 10 µg /cm^2^ may be used as diagnostic concentrations for monitoring Vip3A susceptibility in future. However, additional studies are warranted to identify more specific concentrations for the resistance monitoring. The use of only the 12 populations collected from non-Bt corn during 2018 for estimation of the diagnostic concentrations should minimize any possible effect of recent field selections with Vip3A on the *H. zea* populations. As mentioned above, until recently the use of Vip3A in both transgenic corn and cotton in the U.S. had been very rare. In Brazil, a concentration of 6.4µg Vip3Aa20/cm^2^ has been recommended as a diagnostic concentration for Vip3A susceptibility monitoring in *H. zea* [31], which is within the range of the concentrations suggested in the current study. 

The second generation Bt crops containing pyramided Cry1/Cry2 genes (e.g., Cry1Ac/Cry2Ab for cotton and Cry1A.105/Cry2Ab for corn) became available in 2002 (Bt cotton) and 2010 (Bt corn). These pyramided Cry crop traits initially were effective against *H. zea* [38,39,40]. A few years after their use, unexpected *H. zea* survival and plant damage were frequently observed in the U.S. [1,6,7,8,9]. These unexpected control problems have been documented to be a result of resistance development to Cry1/Cry2 [7,8,9]. Recent studies have shown that the Cry1/Cry2 resistance in *H. zea* has become widely distributed in the southern U.S. [8,9,41,42]. As reported in the reference [26], in the current global Bt crop market, there are only three groups of Bt toxins with different modes of action targeting lepidopteran pests. These are Cry1 (Cry1Ac in cotton, Cry1A.105 in corn, and Cry1Ab and Cry1F in both cotton and corn), Cry2 (Cry2Ae in cotton and Cry2Ab in both cotton and corn), and Vip3A (Vip3Aa19 in cotton and Vip3Aa20 in corn). To date, transgenic corn and cotton traits expressing Vip3A have been effective against *H. zea*, and no field resistance has been documented [21,22,24,43,44]. In the current study, we also conducted massive field surveys (>10,000 ears) for *H. zea* larval survival on corn plants containing Vip3Aa20 gene at multiple locations in Louisiana. The surveys found only a few small live larvae (3rd instars) of *H. zea*. The results of the field surveys also showed no sign of resistance development in the insect to the Vip3Aa20 corn. On the other hand, cross-resistance among Cry1 or Cry2 toxins in lepidopteran species has been well-documented [16,26,45,46,47]. Because of cross-resistance, resistance to one Cry1 or Cry2 toxin will likely result in resistance to all other Cry1 or Cry2 toxins in the plants. Therefore, the widespread Cry1/Cry2 resistance makes Vip3A the only fully active Bt toxin expressed in transgenic plants for controlling *H. zea*. In addition, unexpected control problems with transgenic corn and cotton containing the Vip3A gene have already been reported in the U.S. [1,9]. Therefore, preservation of *H. zea* susceptibility to Vip3A is essential for the continued success of Bt crop technology, especially for the cotton industry in the U.S. where *H. zea* is a key economic pest [1]. Further studies are necessary to understand the impact of the selection with Bt corn on the resistance evolution in *H. zea* to Vip3A cotton in the U.S.

## 4. Conclusions

Resistance to Cry1Ab in *H. zea* is widely distributed in southeastern U.S. Cry1Ab-resistant *H. zea* populations showed no cross-resistance to Vip3Aa20 and the insect in the region is still susceptible to the Vip3Aa20 toxin expressed in MIR 162 corn. A concentration range of 5 to 10 µg Vip3Aa20/cm^2^ may be used as potential diagnostic concentrations in susceptibility monitoring in future. Implementation of effective measures for mitigating the Cry resistance is critical to sustain *H. zea* susceptibility to Vip3A. Additional studies are necessary to elucidate the effect of the selection with Bt corn on resistance evolution in *H. zea* to Vip3A cotton in the U.S.

## 5. Materials and Methods

### 5.1. Insect Sources

During 2018–2019, a total of 32 populations of *H. zea* were established from collections of 3rd to 6th instar larvae from major corn areas in seven southeastern states of the U.S.: 12 populations from Louisiana (LA), 6 from Mississippi (MS), 2 from Arkansas (AR), 2 from Georgia (GA), 6 from South Carolina (SC), 2 from North Carolina (NC), and 2 from Florida (FL) (Figure 1, Table 1 and Table 3). Twenty of the 32 populations were collected during 2018, which consisted of 12 populations from non-Bt corn, 7 from Cry Bt corn, and 1 from WideStrike 3 (Cry/Vip3A) Bt cotton (WS3). The other 12 populations were collected during 2019, which included 7 from non-Bt corn and 5 from Cry corn fields. Among the 12 populations collected from Cry corn during the two years, 10 were collected from Genuity^®^VT Double Pro^TM^ corn (VT2P), 1 from Genuity^®^ SmartStax^®^ corn (SMT), and 1 from Optimum^®^ Intrasect^®^ corn (Intra). VT2P corn produces two pyramided Bt toxins: Cry1A.105 and Cry2Ab2; SMT corn expresses three Bt toxins: Cry1A.105, Cry2Ab2, and Cry1F targeting above-ground lepidopteran pests; Intra corn produces both Cry1Ab and Cry1F; and WS3 cotton expresses Cry1Ac, Cry1F, and Vip3Aa19 [48,49]. Populations collected from a same site used in the study were not necessary from the same spot (fields), but some were from same farms. In addition, massive field surveys (>10,000 ears) for *H. zea* survival on Vip3Aa20 corn were performed at multiple locations in Louisiana during 2018 and 2019. The original purpose of the field surveys was to establish field-collected *H. zea* populations from Vip3Aa20 corn fields for laboratory bioassays with Bt proteins. However, the surveys found only a few small larvae (3^rd^ instars) and thus, populations from Vip3Aa20 corn were not able to be established in this study.

### 5.2. Insect Rearing

Each field insect population originated from collections of 52 to 950 third to sixth instar larvae. Field-collected larvae were individually reared in 30-ml cups containing Ward’s Heliothis meridic diet (Rochester, NY, USA) as described in the reference [8]. Pupae that developed from field-collected larvae for each population were placed into a 20-L mesh cage (Seville Classics, INC., Torrance, CA, USA) and the cages containing pupae were placed in an insect rearing room at 26 °C, >70% R.H. and a photoperiod of 14:10 h (L:D) for adult emergence, mating, and oviposition [8]. Eggs were harvested daily and stored in plastic bags inflated with air. Neonates of the first (F_1_) or second (F_2_) generation of each field-collected population were used in bioassays to determine the susceptibility to Cry1Ab and Vip3Aa20. A laboratory strain of *H. zea* (BZ) obtained from Benzon Research Inc. (Carlisle, PA, USA) was included in the bioassays as a reference. BZ has been documented to be susceptible to several Bt toxins including Cry1A.105, Cry2Ab2, Cry1Ab, and Vip3Aa20 [8,25,41,42].

### 5.3. Diet-Overlay Bioassays 

A diet-overlay bioassay method described in the reference [8] was used to determine the susceptibility of BZ and field-collected populations to Cry1Ab and Vip3Aa20 toxins. Lyophilized Cry1Ab (84.3% purity) and Vip3Aa20 (86.5% purity) toxins were provided by Syngenta Biotechnology (Research Triangle Park, NC, USA). Three (LA-WB-VT2P-1, LA-SJ-VT2P and MS-ST-NBt-2) of the field-collected populations were assayed against Cry1Ab, but not Vip3Aa20; and two populations (AR-LK-VT2P and SC-DL-NBt-2) were tested against Vip3Aa20, but not Cry1Ab. All other 27 field populations were assayed against both Cry1Ab and Vip3Aa20. During the study, three independent bioassays with BZ were conducted for each of the two Bt toxins: two bioassays in 2018 and one in 2019.

Most bioassays for field populations consisted of six or seven Bt concentrations with a range of 0.0316 to 10.0 µg/cm^2^ (six-concentration assay) or 0.01 to 10.0 µg/cm^2^ (seven-concentration assay). For the four populations (LA-WB-VT2P-1, LA-SJ-VT2P, LA-WB-SMT, and FL-SR-VT2P), the highest concentration used in bioassays with Cry1Ab was 31.6 µg/cm^2^. The reason for use of a higher Cry1Ab concentration for these populations was that many populations had exhibited <50% mortality at 10 µg/cm^2^ in earlier bioassays. Each bioassay for BZ utilized 7 to 9 concentrations with a range of 0.000656 to 10.0 µg/cm^2^. To obtain the appropriate toxin concentrations for bioassays, Bt solutions were prepared by mixing lyophilized toxins with distilled water containing 0.1% Triton X-100. Diet treated with distilled water containing 0.1% Triton X-100 only was included as a control for each bioassay. In the bioassays, approximately 0.8 ml of the Southland liquid diet (Lake Village, AR) was applied into each well of 16- or 32-well plates that were made by cutting each 128-well CD-International tray (Pitman, NJ, USA) into four or eight plates. After the diet solidified, 50 µl of an appropriate Bt solution or 0.1% Triton X-100 distilled water only (control) was applied to the diet surface in each well using Eppendorf Repeater^®^ M4 pipettes (Pipett.com, San Diego, CA, USA). The plates were then hand-shaken to ensure uniform coverage of the Bt toxin onto the diet surface [8]. After the Bt solution dried, one *H. zea* neonate (<24 h) was gently placed on the diet surface in each well using a soft hair paint-brush. A bioassay for each combination of insect population and Bt concentration or control utilized four replications (plates) with 16 or 32 larvae in each replicate. The bioassay wells were then covered with BACV16 ventilated lids (CD-International, Pitman, NJ, USA). Bioassay plates containing neonates were maintained in incubators at ~50% R.H., 26°C, and a 16:8h (L:D) photoperiod. Number of real dead larvae and the living larvae that were severely stunted and still in 1st or 2nd instar were recorded 7 days after infection. A larva was considered really dead if the body did not move after touching it with a soft hair paint-brush. In this study, a measurement of ‘practical mortality’ was adopted as the response variable, which was calculated as: larval mortality (%) = {[100 × (number of dead larvae + number of living larvae that were severely stunted and still in 1st or 2nd instar after 7 days in the bioassay)] divided by the total number of larvae assayed} [50]. Studies have reported that the criterion of ‘practical mortality’ is usually more sensitive to measure the responses of insect to Bt toxins than the use of ‘real mortality’ only, because the measurement of ‘practical mortality’ considers both factors including the ‘real mortality’ that is based on ‘real dead larvae’ and the number of the ‘severely stunted larvae’ that is based on larval growth inhabitation [8,50].

### 5.4. Statistical Analysis 

Larval (practical) mortality of *H. zea* populations for each Bt concentration in each dose-response bioassay was corrected for control mortality [51]. The corrected data were analyzed using probit analysis with a normal distribution model (SAS PROC PROBIT) [52,53] to calculate the LC_50_ that resulted in 50% mortality of the population and the associated 95% CI. Dose-responses of the susceptible control, BZ strain, in the three bioassays were consistent, and, thus, mortality data were pooled across bioassays for probit analysis. As mentioned above, in the bioassays with Cry1Ab, some field populations exhibited low mortalities (e.g., <50%) across the concentrations assayed, and thus the corresponding LC_50_ values were not determined with probit analysis. Instead, LC_50_ values for these populations were estimated to be greater than the highest Cry1Ab concentration tested in bioassays (e.g., 10 µg/cm^2^). If the LC_50_ of a field population was greater than that of BZ, relative resistance ratios of the field population were calculated using the LC_50_ value of the field population divided by the LC_50_ of BZ. If the LC_50_ of a field population was less than that of BZ, the susceptibility ratio was calculated using the LC_50_ of BZ divided by the LC_50_ of the field population with a negative sign. In addition, dose-response data from Vip3Aa20 bioassays with the 12 populations collected from non-Bt corn fields during 2018 were pooled. The pooled data were then analyzed using probits to calculate the LC_99_ (the Vip3Aa20 concentration that caused 99% mortality) and its 95% CI. The estimated 95% CIs of the LC_99_ were considered as a potential diagnostic concentration range to identify resistant individuals in field populations of *H. zea*.

As mentioned above, the 13 populations collected from Bt plants during the two years involved crop varieties expressing four different combinations of Bt proteins. Notably, only a single population was collected from each of SMT, Intra, and WS3 crop varieties. In addition, the Cry1 proteins (e.g., Cry1Ab, Cry1Ac, Cry1F, and Cry1A.105) expressed in the Bt crops have similar modes of action and are cross-resistant to each other in lepidopteran species including *H. zea*. Thus, in this study all the populations collected from the four types of Bt crop fields were considered as one group and then Student’s *t*-tests (SAS PROC TTEST) [53] were used to compare the mean susceptibility data of the population group from non-Bt fields. Similarly, t-tests were also conducted to determine if there were significant differences in the Bt susceptibilities between the population groups collected between 2018 and 2019. In the *t*-tests, a population within an insect group was considered as a replication of the group. Based on the results of bioassays with Cry1Ab toxin, the concentration of 10 µg/cm^2^ was chosen to discriminate Cry1Ab-resistant individuals from the insect populations, and, thus, mortality levels observed at 10 µg/cm^2^ were a better estimation of the actual susceptibility to Cry1Ab than the estimated LC_50_ values for those populations with <50% mortality at 10 µg/cm^2^. Thus, for bioassay data with Cry1Ab, larval survivorship (%) of field populations observed at 10 µg/cm^2^ of Cry1Ab was corrected based on the control survival and the corrected mean survivorships at 10 µg/cm^2^ of Cry1Ab were used in the *t*-tests. In contrast, for the bioassays with Vip3Aa20, because LC_50_ values were able to be calculated for almost all the populations assayed, the calculated LC_50_ values were directly compared between the two groups of populations from non-Bt and Bt crops, and between the two groups of populations collected in 2018 and 2009. In addition, linear regressions (SAS PROC REG) [53] were also performed to test if there were significant relationships between the larval survivorships (X) at 10 Cry1Ab µg/cm^2^ and the LC_50_ values (Y) of Vip3Aa20 for the populations collected in 2018 (17 populations), 2019 (9 populations), and the full set of the 26 populations that were assayed against both Cry1Ab and Vip3Aa20 across both years. The reasons for the use of two different statistical criteria for the bioassays with the two Bt proteins in the regression analyses are the same as mentioned in the *t*-tests.

## Figures and Tables

**Table 1:** Susceptibility of *Helicoverpa zea* populations collected from seven southeastern states of the U.S. to Cry1Ab toxin.

Insect Populations ^a^	Collection Locations: County/Parish, State ^b^	Host Sources ^c^	No. Larvae Assayed	Slope ± SE	LC_50_ (95% CI) ^d^ (µg/cm^2^)	χ^2^	*p*-Value	Resistance ratio ^e^	% Larval Survivorship at 10 µg/cm^2^
BZ	Benzon Research Inc.	Laboratory	1699	1.29 ± 0.12	0.21 (0.15, 0.29)	304.09	<0.0001	n/a	0.0 ± 0.0
**Insect populations collected during 2018**									
LA-BR-NBt	East Baton Rouge, LA	Non-Bt corn	324	1.30 ± 0.28	3.51 (2.36, 5.78)	9.20	0.5128	17	26.7 ± 3.7
LA-WB-NBt-1	Franklin, LA	Non-Bt corn	983	0.99 ± 0.18	9.10 (4.78, 29.8)	57.30	<0.0001	43	50.8 ± 8.1
LA-WB-NBt-2	Franklin, LA	Non-Bt corn	368	1.85 ± 0.32	9.10 (6.74, 14.56)	13.00	0.2239	43	50.2 ± 3.1
LA-WB-NBt-3	Franklin, LA	Non-Bt corn	414	1.48 ± 0.30	7.71 (5.30, 10.5)	9.23	0.5106	37	18.6 ± 1.7
LA-WB-VT2P-1	Franklin, LA	VT-2P corn	419	0.93 ± 0.20	14.4 (7.4, 49.0)	36.18	0.0043	69	56.7 ± 4.3
LA-SJ-VT2P	Tensas, LA	VT-2P corn	394	2.81 ± 0.35	12.4 (10.0, 15.3)	15.57	0.1126	59	55.9 ± 5.7
LA-WB-SMT	Franklin, LA	SmartStax corn	255	n/a	>31.6	n/a	n/a	>150	73.0 ± 4.1
LA-GT-WS3	Grant, LA	WideStrike-3 cotton	485	n/a	>10	n/a	n/a	>48	51.8 ± 1.0
MS-ST-NBt-1	Washington, MS	Non-Bt corn	374	0.94 ± 0.12	1.84 (1.27, 2.82)	25.73	0.1062	9	24.2 ± 4.8
MS-LL-NBt	Washington, MS	Non-Bt corn	360	0.78 ± 0.14	2.77 (1.50, 6.88)	28.59	0.0536	13	24.7 ± 5.8
MS-ST-VT2P-1	Washington, MS	VT-2P corn	371	n/a	>10	n/a	n/a	>48	54.7 ± 6.9
MS-LL-Intra	Washington, MS	Intrasect corn	287	0.82 ± 0.24	10.75 (5.76, 64.5)	15.17	0.1261	51	51.8 ± 10.6
GA-TF-NBt-1	Tift, GA	Non-Bt corn	228	n/a	>10	n/a	n/a	>48	74.4 ± 10.2
GA-TF-NBt-2	Tift, GA	Non-Bt corn	439	n/a	>10	n/a	n/a	>48	73.5 ± 6.5
SC-FR-NBt-1	Florence, SC	Non-Bt corn	362	n/a	>10	n/a	n/a	>48	66.9 ± 7.6
SC-FR-NBt-2	Florence, SC	Non-Bt corn	342	n/a	>10	n/a	n/a	>48	82.9 ± 1.6
SC-DL-NBt-1	Darlington, SC	Non-Bt corn	384	n/a	>10	n/a	n/a	>48	59.4 ± 11.0
SC-DL-VT2P-1	Darlington, SC	VT-2P corn	341	0.67 ± 0.19	4.61 (1.84, 38.1)	45.00	0.0004	22	44.1 ± 15.6
NC-CD-NBt	Caldwell, NC	Non-Bt corn	349	1.25 ± 0.20	3.49 (2.39, 5.79)	16.68	0.2736	17	44.2 ± 8.7
FL-SR-VT2P	Jay, FL	VT-2P corn	377	0.79 ± 0.12	1.84 (1.09, 2.97)	16.77	0.2687	9	n/a
**Insect populations collected during 2019**									
LA-WB-NBt-4	Franklin, LA	Non-Bt corn	437	n/a	>10	n/a	n/a	>48	68.7 ± 6.8
LA-AX-NBt	Rapides, LA	Non-Bt corn	441	n/a	>10	n/a	n/a	>48	79.4 ± 6.1
LA-WB-VT2P-2	Franklin, LA	VT-2P corn	478	n/a	>10	n/a	n/a	>48	80.6 ± 5.6
LA-AX-VT2P	Rapides, LA	VT-2P corn	474	0.79 ± 0.11	1.00 (0.60, 1.77)	31.79	0.0810	5	27.0 ± 1.6
MS-ST-NBt-2	Tensas, LA	Non-Bt corn	512	1.02 ± 0.16	1.03 (0.56, 2.09)	84.87	<0.0001	5	14.6 ± 4.0
MS-ST-VT2P-2	Tensas, LA	VT-2P corn	447	0.75 ± 0.11	2.32 (1.48, 4.11)	24.70	0.1334	11	37.9 ± 8.2
AR-TR-NBt	Desha, AR	Non-Bt corn	510	0.83 ± 0.09	0.46 (0.31, 0.68)	19.87	0.5913	2	9.7 ± 1.9
SC-DL-VT2P-2	Darlington, SC	VT-2P corn	511	0.65 ± 0.11	5.99 (3.24, 16.54)	21.74	0.2438	29	34.9 ± 6.6
NC-HA-NBt	Harnett, NC	Non-Bt corn	506	1.10 ± 0.18	8.36 (5.43, 16.59)	13.36	0.4981	40	50.8 ± 9.3
FL-SR-NBt	Jay, FL	Non-Bt corn	502	1.15 ± 0.25	0.40 (0.19, 0.72)	33.50	0.0024	2	3.9 ± 3.9

^a^ BZ was a known Bt-susceptible laboratory strain; ^b^ LA = Louisiana, MS = Mississippi, AR = Arkansas, GA = Georgia, SC = South Carolina, NC = North Carolina, and FL = Florida; ^c^ ‘VT2P’, ‘SMT’, ‘Intra’ or ‘WS3’ indicates that the population was sampled from Genuity^®^ VT Double^®^ PRO Bt corn, Genuity^®^SmartStax^®^ Bt corn, Intrasect^®^ Bt corn, or WideStrike^®^ 3 Bt cotton fields, respectively. The VT2P corn trait contains the event MON 89034 expressing the pyramided Cry1A.105/Cry2Ab2 toxins; SMT contains MON 89034 and TC1507 (Cry1F); Intra produces Cry1Ab and Cry1F; and WS3 cotton contains three pyramided Bt toxins: Cry1Ac, Cry1F and Vip3Aa19; ^d^ The highest Bt concentrations used in the bioassays was 31.6 µg/cm^2^ for LA-WB-VT2P-1, LA-SJ-VT2P, LA-WB-SMT, and FL-SR-VT2P, while it was 10 µg/cm^2^ for all other populations. Larval mortalities of some field-collected populations were low (e.g., <50%) even at the highest Bt concentrations assayed. The LC_50_s for these populations were considered to be greater than the highest concentrations used in the bioassay; ^e^ Resistance ratio of a field population to Cry1Ab was computed by dividing the LC_50_ value of the population by the LC_50_ of BZ; n/a: data not available.

**Table 2:** Comparisons of the mean survivorships at 10 µg/cm^2^ of Cry1Ab between the two groups of *Helicoverpa zea* populations collected from non-Bt and Bt crop fields, and between the two groups of populations sampled during 2018 and 2019.

Insect Population Groups	Mean Survivorship ± SEM%	*t*-Tests
12 populations collected from non-Bt fields in 2018	49.7 ± 6.4	
7 populations collected from Bt fields in 2018	55.4 ± 3.3	*t*_17_ = −0.64, *p* = 0.5284
6 populations collected from non-Bt fields in 2019	37.9 ± 13.3	
4 populations collected from Bt fields in 2019	45.1 ± 12.1	*t*_8_ = −0.38, *p* = 0.7155
19 populations collected from non-Bt and Bt fields in 2018	51.8 ± 4.2	
10 populations collected from non-Bt and Bt fields in 2019	40.8 ± 8.9	*t*_27_ = 1.28, *p* = 0.2125
18 populations collected from non-Bt fields in 2018 and 2019	45.8 ± 6.1	
11 populations collected from Bt fields in 2018 and 2019	51.7 ± 4.7	*t*_27_ = −0.68, *p* = 0.5006

**Table 3:** Susceptibility of *Helicoverpa zea* populations collected from seven southeastern states of the U.S. to Vip3Aa20 toxin.

Insect Populations ^a^	Collection Locations: County/Parish, State ^b^	Host Sources ^c^	No. Larvae Assayed	Slope ± SE	LC_50_ (95%CI) (µg/cm^2^)	χ^2^	*p*-Value	Susceptibility Ratio ^d^
BZ	Benzon Research, Inc.	Laboratory	1652	2.47 ± 0.39	0.40 (0.30, 0.51)	113.0	<0.0001	-
**Insect populations collected during 2018**								
LA-BR-NBt	East Baton Rouge, LA	Non-Bt corn	365	4.41 ± 0.60	0.20 (0.17, 0.23)	12.49	0.2535	−2.0
LA-WB-NBt-1	Franklin, LA	Non-Bt corn	1004	2.43 ± 0.24	0.96 (0.78, 1.12)	23.68	0.0501	2.4
LA-WB-NBt-2	Franklin, LA	Non-Bt corn	383	2.14 ± 0.54	0.13 (0.06, 0.22)	50.73	<0.0001	–3.1
LA-WB-NBt-3	Franklin, LA	Non-Bt corn	367	1.92 ± 0.23	0.21 (0.16, 0.27)	9.85	0.7730	−1.9
LA-WB-SMT	Franklin, LA	SmartStax corn	350	2.19 ± 0.23	0.37 (0.26, 0.41)	14.19	0.7163	−1.1
LA-GT-WS3	Grant, LA	WideStrike-3 cotton	370	1.71 ± 0.25	0.92 (0.59, 1.31)	22.11	0.0763	2.3
MS-ST-NBt-1	Washington, MS	Non-Bt corn	362	1.43 ± 0.24	0.32 (0.17, 0.51)	43.94	0.0006	−1.3
MS-LL-NBt	Washington, MS	Non-Bt corn	343	1.89 ± 0.47	0.14 (0.05, 0.23)	45.11	<0.0001	−2.9
MS-ST-VT2P-1	Washington, MS	VT-2P corn	375	3.16 ± 0.48	0.48 (0.36, 0.65)	30.40	0.0067	1.2
MS-LL-Intra	Washington, MS	Intrasect corn	358	1.96 ± 0.26	0.46 (0.32, 0.66)	32.91	0.0116	1.2
GA-TF-NBt-1	Tift, GA	Non-Bt corn	369	2.34 ± 0.26	0.42 (0.34, 0.51)	10.86	0.9003	1.1
GA-TF-NBt-2	Tift, GA	Non-Bt corn	446	n/a	<0.0316	n/a	n/a	<−11.5
SC-FR-NBt-1	Florence, SC	Non-Bt corn	368	2.55 ± 0.41	0.30 (0.21, 0.41)	35.75	0.0049	−1.3
SC-FR-NBt-2	Florence, SC	Non-Bt corn	365	2.22 ± 0.24	0.34 (0.27, 0.42)	11.61	0.6379	−1.2
SC-DL-NBt-1	Darlington, SC	Non-Bt corn	438	4.91 ± 0.59	0.88 (0.78, 1.01)	4.90	0.9872	2.2
SC-DL-VT2P-1	Darlington, SC	VT-2P corn	367	2.05 ± 0.33	0.31 (0.21, 0.45)	29.69	0.0084	−1.3
NC-CD-NBt	Caldwell, NC	Non-Bt corn	369	1.79 ± 0.17	0.48 (0.37, 0.61)	23.69	0.1656	1.2
FL-SR-VT2P	Jay, FL	VT-2P corn	376	2.46 ± 0.68	0.17 (0.07, 0.28)	90.42	<0.0001	−2.4
**Insect populations collected during 2019**								
LA-WB-NBt-4	Franklin, LA	Non-Bt corn	511	2.27 ± 0.21	0.25 (0.20, 0.30)	14.66	0.6853	−1.6
LA-AX-NBt	Rapides, LA	Non-Bt corn	497	2.57 ± 0.26	1.34 (1.10, 1.63)	17.79	0.2165	3.4
LA-WB-VT2P-2	Franklin, LA	VT-2P corn	256	-	<0.316	-	-	<1.3
LA-AX-VT2P	Rapides, LA	VT-2P corn	512	2.98 ± 0.30	0.06 (0.05, 0.07)	24.04	0.1539	−6.7
MS-ST-VT2P-2	Washington, MS	VT-2P corn	512	1.49 ± 0.14	0.21 (0.16, 0.28)	21.70	0.2455	−1.9
AR-TR-NBt	Desha, AR	Non-Bt corn	500	1.21 ± 0.18	0.04 (0.02, 0.06)	33.71	0.0137	−10.0
AR-LK-VT2P	Lonoke, AR	VT-2P corn	507	1.86 ± 0.21	3.60 (2.84, 4.69)	6.51	0.9520	9.0
SC-DL-NBt-2	Darlington, SC	Non-Bt corn	1015	1.95 ± 0.19	1.32 (1.03, 1.71)	93.73	<0.0001	3.3
SC-DL-VT2P-2	Darlington, SC	VT-2P corn	511	1.25 ± 0.13	0.65 (0.49, 0.96)	15.31	0.6409	1.6
NC-HA-NBt	Harnett, NC	Non-Bt corn	511	3.07 ± 0.33	0.08 (0.06, 0.09)	18.20	0.1977	−5.0
FL-SR-NBt	Jay, FL	Non-Bt corn	509	1.37 ± 0.14	0.12 (0.09. 0.17)	41.34	0.0287	−3.3

^a^ BZ was a known Bt-susceptible laboratory strain; ^b^ LA = Louisiana, MS = Mississippi, AR = Arkansas, GA = Georgia, SC = South Carolina, NC = North Carolina, and FL = Florida; ^c^ ‘VT2P’, ‘SMT’, ‘Intra’ or ‘WS3’ indicates that the population was sampled from Genuity^®^ VT Double^®^ PRO Bt corn, Genuity^®^SmartStax^®^ Bt corn, Intrasect® Bt corn, or WideStrike^®^ 3 Bt cotton fields, respectively. The VT2P corn trait contains the event MON 89034 expressing the pyramided Cry1A.105/Cry2Ab2 toxins; SMT contains MON 89034 and TC1507 (Cry1F); Intra produces Cry1Ab and Cry1F; and WS3 cotton contains three pyramided Bt toxins: Cry1Ac, Cry1F and Vip3Aa19; ^d^ If LC_50_ of a field population was greater than that of BZ, relative susceptibility ratio of the field population was calculated using the LC_50_ value of the field population divided by LC_50_ of BZ; otherwise, if LC_50_ of a field population was less than that of BZ, its susceptibility ratio was calculated using LC_50_ of BZ divided by LC_50_ of the field population with a negative sign; n/a: data not available.

**Table 4:** Comparisons of the mean LC_50_ values of Vip3Aa20 between the two groups of *Helicoverpa zea* populations collected from non-Bt and Bt crop fields, and between the two groups of populations sampled during 2018 and 2019.

Insect Population Groups	Mean LC_50_ ± SEM (µg/cm^2^)	*t*-Tests
12 populations collected from non-Bt fields in 2018	0.37 ± 0.08	
6 populations collected from Bt fields in 2018	0.45 ± 0.10	*t*_17_ = −0.60, *p* = 0.5540
6 populations collected from non-Bt fields in 2019	0.53 ± 0.26	
5 populations collected from Bt fields in 2019	0.97 ± 0.67	*t*_8_ = −0.67, *p* = 0.5221
18 populations collected from non-Bt and Bt fields in 2018	0.42 ± 0.06	
11 populations collected from non-Bt and Bt fields in 2019	0.73 ± 0.32	*t*_27_ = −1.26, *p* = 0.2175
18 populations collected from non-Bt fields in 2018 and 2019	0.42 ± 0.10	
11 populations collected from Bt fields in 2018 and 2019	0.69 ± 0.30	*t*_27_ = −1.01, *p* = 0.3234

## Data Availability

Not applicable.
